# Yankees in Russia

**Published:** 1881-07

**Authors:** 


### YANKEES IN RUSSIA.

HATING the Germans, looking down on the French, disliking the English, the Russians seem to reserve all their good will for Americans. With them "American" or "Yankee" is the synonym of indomitable energy, of intrepid enterprise, of wonderful ability, and of frank truth-telling. They call their own ablest engineers, inventors and discoverers "Yankees" by way of compliment. M. Jablochkoff, the inventor of the electric candle, and Col. Prejevalsky, the bold Asiatic explorer, are sample Russian Yankees.

Once I was talking with a Russian friend about the possible destiny of our globe. "There is no need of worrying ourselves about the fate of the globe," he said, "for there can be no serious danger for her so long as she has on board our transatlantic friends. If a collision with some other planet should threaten her, the Yankees would at once rig up a rudder, sails, or some other device, and get her out of the scrape."

The Russians are particularly charmed with the democratic manners of Americans. These appeal to a characteristic national trait of the Russians themselves. They despise from the bottom of their hearts all pretension, arrogance, and walking on stilts. That is why the Russians stretch friendly hands to the people across the ocean, in spite of the abyss that lies between their government and that of the Union.

My personal experience is that American citizens in general, and American business men in particular, are warmly welcomed in Russia. On the part of the Czar's government there is not the least fear that they will inoculate the Russians with republicanism. Once I asked a colonel of gendarmes whether he had any apprehension of dangerous results from the close relations of the Russians and the Americans. "Not the least," he answered promptly. "Your citizens are too sensible and practical to be dangerous to our government. To imagine a practical Yankee indulging in theorizing with idle Russians would be to suppose the most improbable of all improbable things."

The Russian capitalists and business men in general are apparently glad to have Americans come here, and closely observe their ways of doing business. They prefer to invite American engineers to Russia instead of sending their engineers to study in America.—*St. Petersburg Letter.*

---

Moderate exercise is one of the conditions of good health.

### HOW TO TRAIN THE MEMORY.

YOUR memory is bad, perhaps, but there are two ways of curing the worst memory. One of them is to read a subject when interested; the other is not only to read but think. When you have read a paragraph or a page stop, close the book, and try to remember the ideas on the page, and not only call them vaguely to mind but put them in words and speak them out. Faithfully following these two rules, and you have the golden keys of knowledge. Beside inattentive reading, there are other things injurious to the memory. One is the habit of skimming over newspapers, items of news, smart remarks, items of information, political reflections, fashion notes, so that all is a confused jumble, never to be thought of again, thus diligently cultivating a habit of careless reading hard to break. Another is the reading of trashy novels.

